# Cryoablation Versus Radiofrequency Ablation for Hepatic Malignancies: A Systematic Review and Literature-Based Analysis

**DOI:** 10.1097/MD.0000000000002252

**Published:** 2015-12-11

**Authors:** Shunquan Wu, Jun Hou, Yingying Ding, Fuquan Wu, Yan Hu, Qiyu Jiang, Panyong Mao, Yongping Yang

**Affiliations:** From the Research Center for Clinical and Translational Medicine, the 302nd Hospital of PLA, Beijing, China (SW, JH, YH, QJ, PM); Department of Medical Microbiology and Parasitology, Second Military Medical University, Shanghai, China (YD); Department of General Surgery, the 309th Hospital of PLA, Beijing, China (FW); Center of Therapeutic Research of Hepatocellular Carcinoma, the 302nd Hospital of PLA, Beijing, China (YY)

## Abstract

The aim of this study is to summarize and quantify the current evidence on the therapeutic efficacy of cryoablation compared with radiofrequency ablation (RFA) in patients with hepatic malignancies in a meta-analysis.

Data were collected by searching PubMed, Scopus, and Cochrane databases for reports published up to May 26, 2015. Studies that reported data on comparisons of therapeutic efficacy of cryoablation and RFA were included. The random effects model was used to estimate the pooled relative risks of events comparing cryoablation to RFA for therapy of hepatic malignancies.

Seven articles met the inclusion criteria and were included in the meta-analysis. The meta-analysis showed that there was no statistically significant difference in mortality of at least 6 months (odds ratio [OR] = 1.00, 95% confidence interval [CI]: 0.68–1.49) and local tumor progression according to both patients (OR = 1.64, 95% CI: 0.57–4.74) and tumors (OR = 1.81, 95% CI: 0.74–4.38) between cryoablation group and RFA group. However, the risk of complications was significantly higher in the cryoablation group than that in the RFA group (OR = 2.93, 95% CI: 1.15–7.46). When considering the specific complications, only thrombocytopenia (OR = 51.13, 95% CI: 2.92–894.21) and renal impairment (OR = 4.19, 95% CI: 1.34–13.11) but not other complications were significantly higher in the cryoablation group.

In conclusion, the 2 methods had almost equal mortality and nonsignificant difference in local tumor progression, with higher risk of complications in cryoablation. Further large-scale, well-designed randomized controlled trials are needed to identify the current findings and investigate the long-term effects of cryoablation compared with RFA for therapy of hepatic malignancies.

## INTRODUCTION

Hepatocellular carcinoma (HCC) is the sixth most common cancer and the third most common cause of cancer-related death worldwide.^1^ The liver is second only after lymph nodes as a common site of metastasis from other solid cancers.^2^ Surgical resection with curative intent remains the optimal treatment for HCC and liver metastases (METS). However, only 5% to 15% patients with HCC and less than 10% to 15% of patients with liver-only solid tumor metastases are candidates for resection.

Other techniques of therapy have been explored for patients who are not suitable for hepatic resection. Local tumor ablative techniques, such as cryoablation and radiofrequency ablation (RFA), may offer an alternative treatment option for those with unresectable hepatic malignancies. Cryoablation is a long known ablative technique which can lead to protein denaturation and cellular dehydration due to the application of extreme low temperatures to tumor tissue.^3,4^ Cryoablation was reported to decrease the mortality in several studies,^5–8^ but it can lead to complications that associated with multiple freezes. RFA has been the most widely utilized percutaneous ablative technology in the liver,^9^ which is based on the principle of generating heat on the tumor tissue.^10–12^ It has been reported to be not only effective but also safe in the treatment of small hepatic malignant neoplasms.^10,11,13^

Potential advantages of cryoablation relative to RFA include adequate tumor coverage while avoiding excessively large ablation volumes or propagation into adjacent critical structures and less diaphragmatic injury and post procedural pain when treating hepatic dome tumors.^14–16^ However, data are scarce comparing the outcomes of cryoablation and RFA, especially from a randomized controlled trial (RCT). A comprehensive meta-analysis remains the most appropriate means to make a comparison between cryoablation and RFA. Our objective was to conduct a meta-analysis to summarize and quantify the current evidence on the therapeutic efficacy of cryoablation compared with RFA in patients with hepatic malignancies.

## MATERIALS AND METHODS

### Literature Search, Study Selection, and Data Extraction

This systematic review and meta-analysis follows the Meta-analysis of Observational Studies in Epidemiology (MOOSE) group (Table S1).^17^ Two researchers (FW and JH) systematically searched PubMed, Scopus, and Cochrane databases for reports published up to May 26, 2015, using a combined text and MeSH heading search strategy with the terms: “cryosurgery ablation,” “cryoablation,” “radiofrequency ablation,” “radio frequency ablation,” “hepatocellular cancer,” “liver,” “liver tumor,” “liver cancer,” “liver neoplasms,” “hepatic tumor,” “metastases,” and “metastasis.” The search was restricted to studies in human beings that were published in or translated into English. We also checked the reference lists of identified reports for other potentially relevant studies. We included studies that met the following criteria: participants aged 18 years or older; prospective design, retrospective design, or randomized controlled design; masked assessment of outcomes; recorded data on results of therapy of cryoablation and RFA (mortality, local tumor progression, and/or complications); and reported data on relative risks (RRs) or odds ratios (ORs) with confidence intervals (CIs) or sufficient information to calculate these, for the association between cryoablation and RFA for therapy of hepatic malignancies. Studies were excluded if they did not provide information to calculate the point estimate, did not make comparison between cryoablation and RFA, or were review studies.

When duplicate reports from the same study were identified, only the most recent publication, or the one with the longest follow-up period, was included. Full text of the article was reviewed if it cannot be excluded by initial review. Two reviewers (YD and FW) extracted the characteristics of each included study, including author, region, study design, disease type, treatment methods, number of participants, number of events (mortality, local tumor progression, and/or complications), percentage of male gender, mean age of participants, mean follow-up duration, mean tumor size, tumor number, and factors balanced. Primary author was contacted for additional information. Institutional review board approval and patient consent were not required for this meta-analysis of observational studies.

### Statistical Analysis

The random effects model was used to estimate the pooled RRs of events comparing cryoablation to RFA for therapy of hepatic malignancies to take into account heterogeneity among studies, since the study design and measuring time were varied across studies. The χ^2^ test and I^2^ statistic were used to assess the percentage of variability attributable to heterogeneity beyond chance across studies.^18^*P* > 0.10 for the χ^2^ test and I^2^ < 25% were interpreted as signifying low-level heterogeneity. Subgroup analyses were performed according to the geographic location (The United States, Europe, or China), mean age of participants (<60 years or ≥60 years), study design (prospective study, retrospective study, or RCT), publication year (pre-2005 or 2005 onwards), disease type (HCC alone, METS alone, or HCC and METS combined), and other factors balanced, to test the possible impact factors. We also performed sensitivity analyses by removing each individual study from the meta-analysis.^19^ Funnel plots were used to examine the presence of publication bias (ie, by plotting the natural log of the OR against its standard error). We also used Egger regression test^20^ and Begg–Mazumdar test^21^ to further assess publication bias. Statistical significance was defined as a 2-tailed *P* < 0.05. All statistical analyses were conducted with RevMan, version 5, from the Cochrane Collaboration (http://www.cochrane.org/).

## RESULTS

### Study Characteristics

**FIGURE 1:**
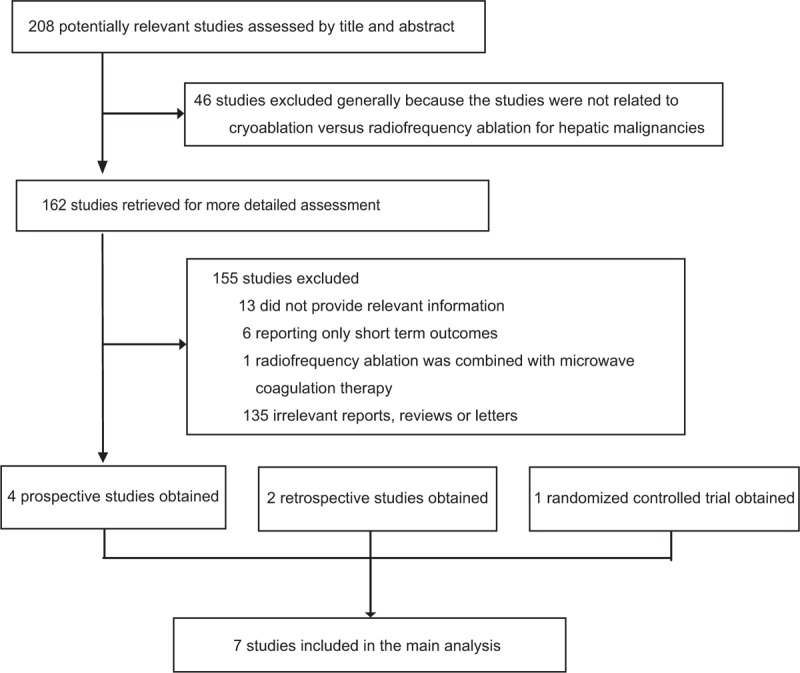
Flowchart for the selection of eligible studies.

The systematic search identified 208 potentially relevant articles, which were assessed by title and abstract. Of these, 162 articles were qualified for selection (Figure 1). After full-text assessment, a total of 7 articles met the inclusion criteria and were included in the meta-analysis, including 4 prospective studies,^22–25^ 2 retrospective studies,^26,27^ and 1 RCT.^28^ Primary characteristics of the 7 included studies are provided in Table 1. Among the included studies, 3 were from Europe, 3 were from The United States, and 1 was from China. Overall, data were available from 1029 patients with hepatic malignancies, of whom 577 were treated with cryoablation and 452 were treated with RFA. Three studies involved patients with HCC, 1 involved patients with METS, and 3 involved patients with both HCC and METS.

**TABLE 1:**
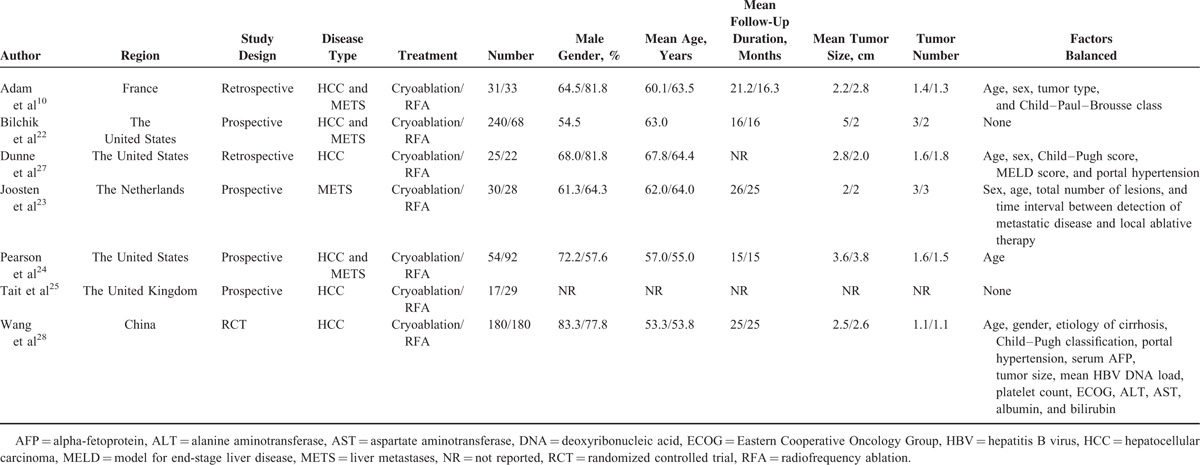
Characteristics of Included Studies

### Mortality

**FIGURE 2:**
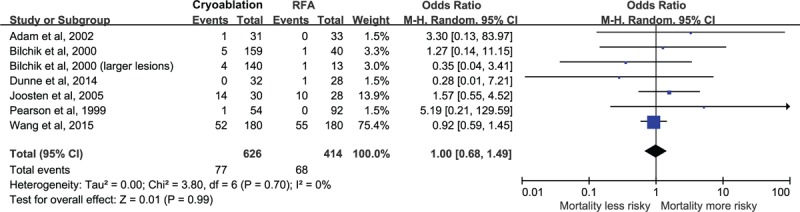
Relative risk of mortality of at least 6 months, comparing patients in the cryoablation group to those in the RFA group.

Data on mortality of at least 6 months were available for analysis in 626 patients in the cryoablation group with 77 deaths and 414 patients in the RFA group with 68 deaths. The meta-analysis showed that there was no statistically significant difference in mortality of at least 6 months between cryoablation group and RFA group (OR = 1.00, 95% CI: 0.68–1.49) (Figure 2). There was no evidence of heterogeneity among individual studies (*P* = 0.70, I^2^ = 0%). We observed that the study of Wang et al^28^ accounted for a large weight (75.4%). Therefore, we pooled the results again by omitting this study, and the OR was not materially changed (OR = 1.29, 95% CI: 0.59–2.86). In the subgroup analyses, the pooled ORs did not differ significantly by geographic location, mean age of participants, study design, publication year, disease type, and other factors balanced or not (Table 2 ). Sensitivity analysis indicated that the nonsignificant difference in mortality was not materially changed in the leave-one-out analyses by omitting 1 study in turn, with pooled ORs range from 0.93 (95% CI: 0.61–1.41) to 1.28 (95% CI: 0.58–2.83), comparing patients in the CSA group to those in the RFA group.

**TABLE 2:**
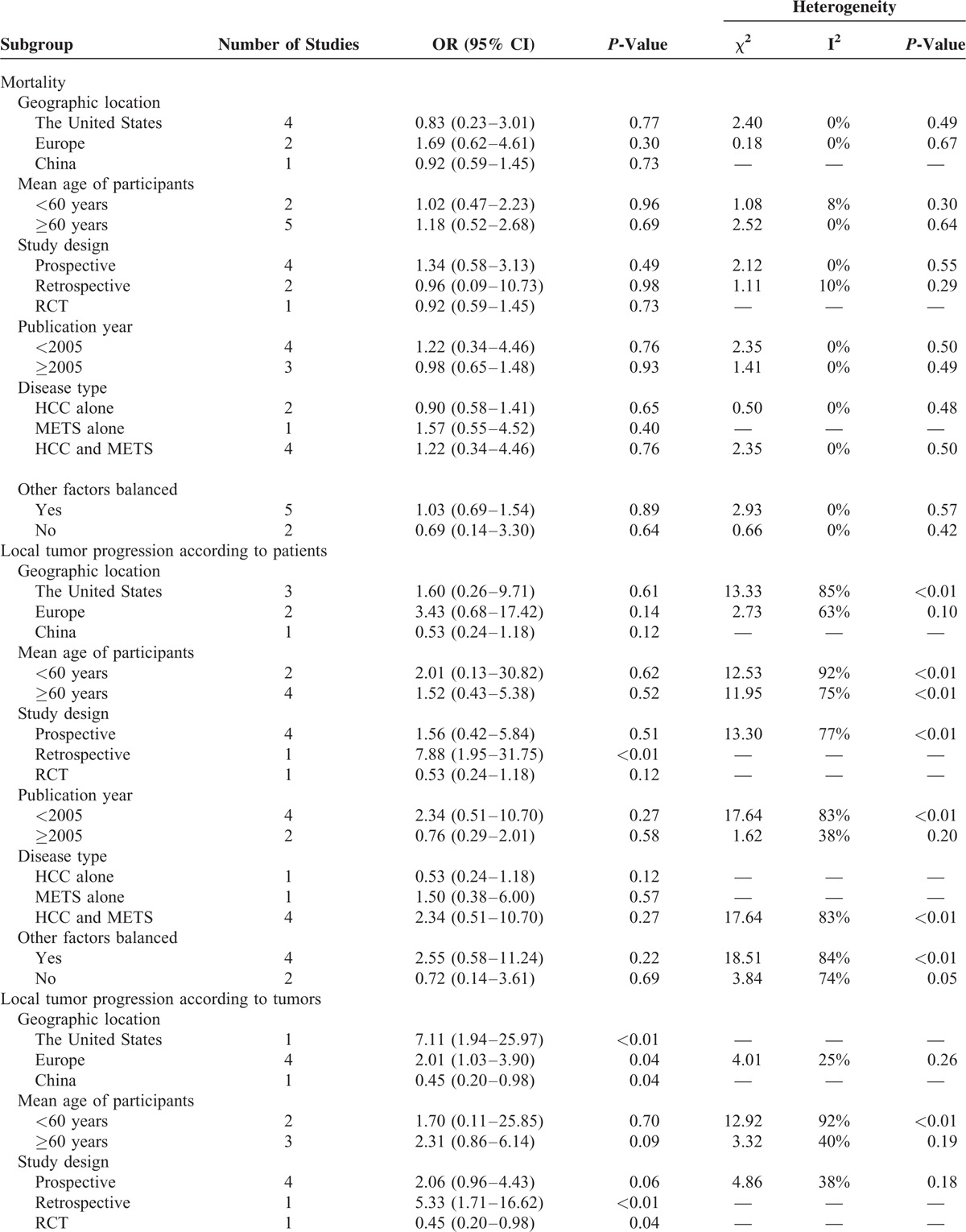
Subgroup Analyses, Comparing Patients in the Cryoablation Group to Those in the RFA Group

**TABLE 2 (Continued):**
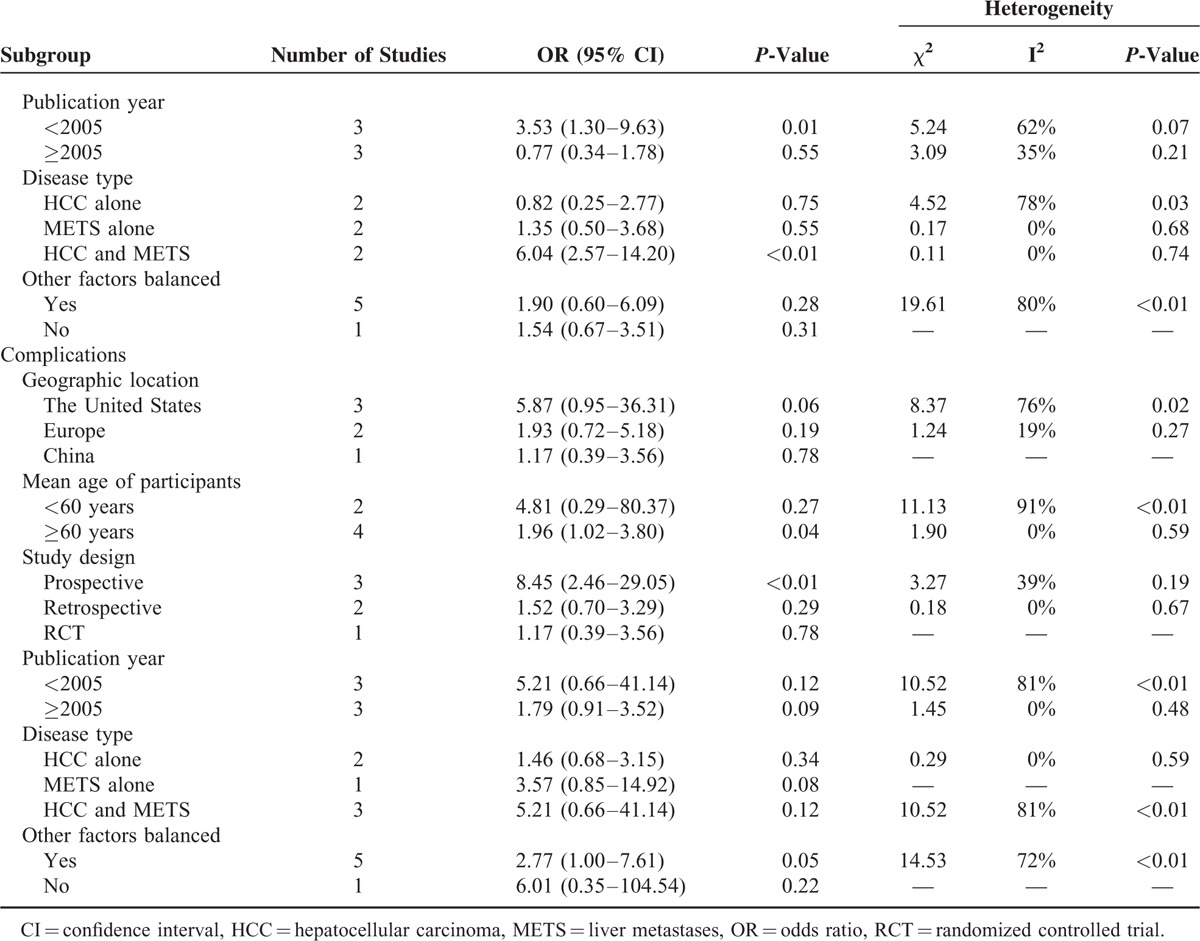
Subgroup Analyses, Comparing Patients in the Cryoablation Group to Those in the RFA Group

### Local Tumor Progression

**FIGURE 3:**
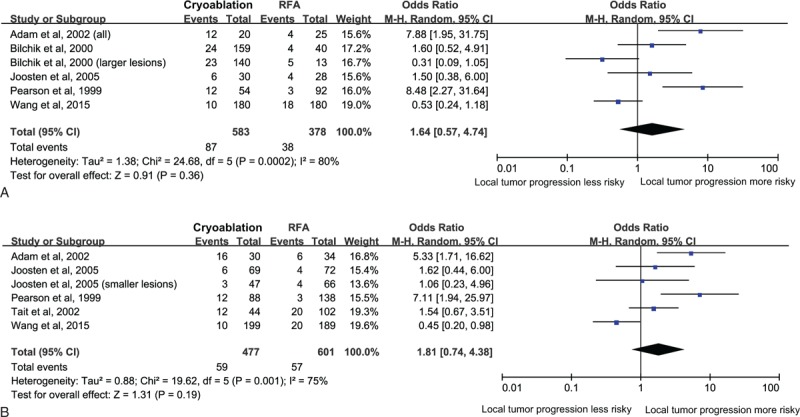
Relative risk of local tumor progression according to patients (A) and relative risk of local tumor progression according to tumors (B), comparing patients in the cryoablation group to those in the RFA group.

Data on local tumor progression according to patients were available for analysis in 583 patients in the cryoablation group with 87 events and 378 patients in the RFA group with 38 events. The result of meta-analysis indicated that there was no statistically significant difference in local tumor progression according to patients between cryoablation group and RFA group (OR = 1.64, 95% CI: 0.57–4.74) (Figure 3A). The I^2^ statistic for heterogeneity between studies was 80%, with *P*-value for the χ^2^ test 0.0002, suggesting substantial between-study heterogeneity. In the subgroup analyses, the pooled ORs did not differ significantly by most of the study-level factors except for study design (Table 2 ). In the subgroup analysis of retrospective study, the RR of local tumor progression according to patients was significantly higher in patients treated with cryoablation than those treated with RFA (OR = 7.88, 95% CI: 1.95–31.75), based on only 1 study. Additionally, sensitivity analysis indicated that the nonsignificant difference in local tumor progression according to patients was not materially changed in the leave-one-out analyses by omitting 1 study in turn, with pooled ORs range from 1.18 (95% CI: 0.43–3.24) to 2.27 (95% CI: 0.74–7.00), comparing patients in the cryoablation group to those in the RFA group.

Data on local tumor progression according to tumors were available for analysis in 477 patients in the cryoablation group with 59 events and 601 patients in the RFA group with 57 events. Similarly, meta-analysis did not show significant difference in local tumor progression according to tumors between cryoablation group and RFA group (OR = 1.81, 95% CI: 0.74–4.38) (Figure 3B). Potential heterogeneity was explored among the individual studies (*P* = 0.001, I^2^ = 75%). Subgroup analyses showed that the RR of local tumor progression according to tumors was significantly higher in patients treated with cryoablation than those treated with RFA in studies that conducted in The United States (OR = 7.11, 95% CI: 1.94–25.97) and Europe (OR = 2.01, 95% CI: 1.03–3.90), in 1 study with retrospective design (OR = 5.33, 95% CI: 1.71–16.62), in studies published before 2005 (OR = 3.53, 95% CI: 1.30–9.63), and in studies that involved both HCC and METS patients (OR = 6.04, 95% CI: 2.57–14.20) (Table 2 ). Sensitivity analysis indicated that the nonsignificant difference in local tumor progression according to tumors was not materially changed in the leave-one-out analyses by omitting 1 study in turn except for the study of Wang et al,^28^ with pooled ORs range from 1.40 (95% CI: 0.59–3.31) to 2.52 (95% CI: 1.24–5.11).

### Complications

**FIGURE 4:**
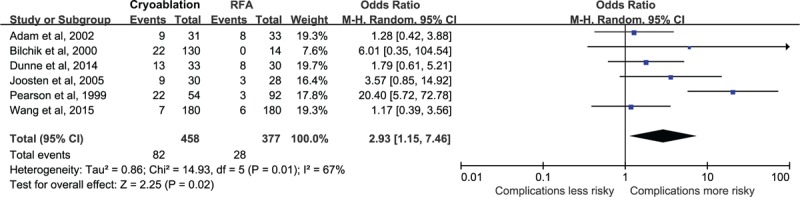
Relative risk of complications, comparing patients in the cryoablation group to those in the RFA group.

Data on complications after therapy were available for analysis in 458 patients in the cryoablation group with 82 events and 377 patients in the RFA group with 28 events. The pooled analysis showed that the risk of complications was significantly higher in the cryoablation group, compared with the RFA group (OR = 2.93, 95% CI: 1.15–7.46) (Figure 4). There was potential heterogeneity among the individual studies (*P* = 0.01, I^2^ = 67%). However, subgroup analyses indicated that the significantly higher risk was only seen in patients with mean age over 60 years (OR = 1.96, 95% CI: 1.02–3.80), in studies with prospective design (OR = 8.45, 95% CI: 2.46–29.05), and in studies that balanced other factors (OR = 2.77, 95% CI: 1.00–7.61) (Table 2 ). The leave-one-out analyses indicated that there was no significant difference in complications between cryoablation group and RFA group when omitting the study of Joosten et al^23^ (OR = 2.85, 95% CI: 0.94–8.68) or the study of Pearson et al^24^ (OR = 1.71, 95% CI: 0.97–3.02).

**FIGURE 5:**
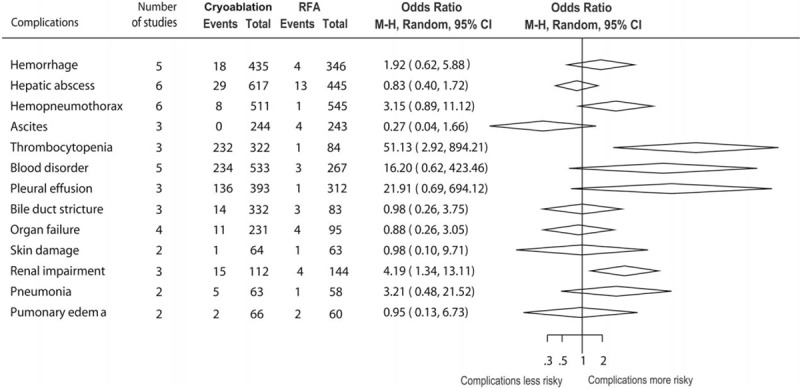
Summary of relative risks of some specific complications, comparing patients in the cryoablation group to those in the RFA group.

We also made summary of RRs of several specific complications, comparing patients in the cryoablation group to those in the RFA group. Although the overall estimate showed that the risk of total complications was significantly higher in the cryoablation group, there was no significant difference in most specific complications between cryoablation group and RFA group (Figure 5). The cryoablation therapy was only associated with a significant increase in the OR for thrombocytopenia (OR = 51.13, 95% CI: 2.92–894.21) and renal impairment (OR = 4.19, 95% CI: 1.34–13.11), compared with the RFA therapy.

### Publication Bias

**FIGURE 6:**
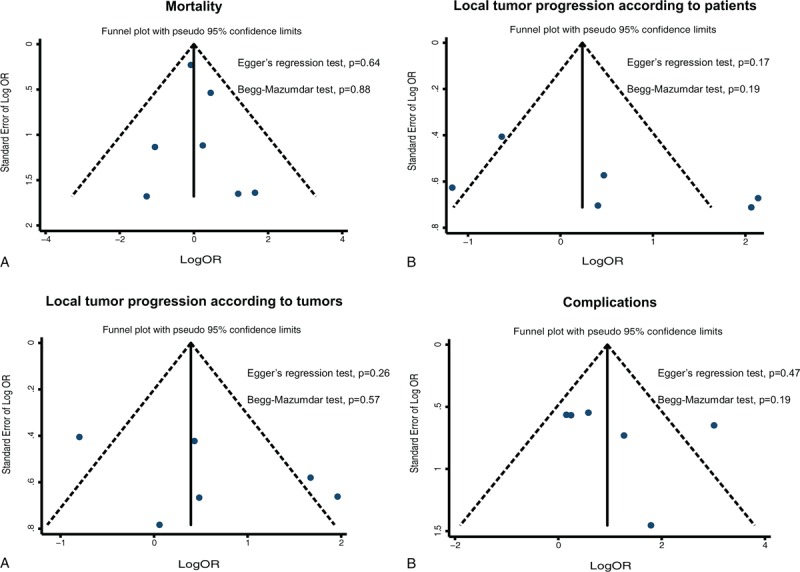
Funnel plots to explore publication bias in the estimates of mortality (A), local tumor progression according to patients (B), local tumor progression according to tumors (C), and complications (D). The vertical line is at the mean effect size.

There was no potential publication bias in all the meta-analysis, as assessed by funnel plots (Figure 6), Egger regression test and Begg–Mazumdar test (all *P* values > 0.05).

## DISCUSSION

Findings from the meta-analysis of 7 studies indicate that there were no significant differences in mortality of at least 6 months and local tumor progression according to both patients and tumors between cryoablation group and RFA group. However, patients in the cryoablation group had significantly higher risk of complications than those in the RFA group. When considering the specific complications, only the risks of thrombocytopenia and renal impairment but not other complications were significantly higher in the cryoablation group, compared with the RFA group.

There is still a lack of RCTs to directly compare the treatment effects and safety profile between cryoablation and RFA for therapy of unresectable hepatic malignancies. Wang et al^28^ conducted the first prospective, multicenter RCT to compare cryoablation with RFA on their clinical outcomes in treating HCC patients in the Chinese population. This first RCT was important since RCTs have been accepted as the golden standard to determine the effectiveness of the intervention, making results “evidence based.” However, a comprehensive meta-analysis is still needed to compare cryoablation with RFA to compensate for the individual lack of precision in most of the published studies, a problem that could be alleviated by pooling the data of all the studies. Therefore, meta-analysis of previous studies is a potentially powerful approach to evaluate the long-term effects of cryoablation compared with RFA in patients with hepatic malignancies.

In our meta-analysis, the point estimate of RR for mortality of at least 6 months was 1.00, comparing patients in the cryoablation group to those in the RFA group with pooled rates of 12.3% (77 of 626) and 16.4% (68 of 414), which suggests that the 2 methods are equally efficient for initial treatment success. In addition, there were no significant differences in local tumor progressions between 2 groups, although the point estimates were over 1. Overall, the local tumor progression following cryoablation was 14.9% (87 of 583) of patients and 12.4% (59 of 477) of tumors compared with 10.1% (38 of 378) of patients and 9.5% (57 of 601) of tumors following RFA. The pooled rates of local tumor progressions in our meta-analysis were in the interval of those reported in previous studies specifically dedicated to cryoablation (2.3–44.0%)^29–32^ or to RFA (1.8–18.0%).^2,33–35^ In the subgroup analyses, the risks of local tumor progressions of cryoablation compared with RFA were shrinked following treatment for HCC alone with point estimates below 1 (OR = 0.53, 95% CI: 0.24–1.18 according to patients, and OR = 0.82, 95% CI: 0.25–2.77 according to tumors), and were amplified following treatment for HCC and METS combined with point estimates increasing (OR = 2.34, 95% CI: 0.51–10.70 according to patients, and OR = 6.04, 95% CI: 2.57–14.20 according to tumors). This means when treating for HCC alone, the risks of local tumor progression was relatively lower in the cryoablation group than those in the RFA group, and when patients with METS involved, the risks of local tumor progression was relatively higher in the cryoablation group than those in the RFA group. This is consistent with previous findings that metastatic tumors treated by cryoablation tend to have a higher local tumor progression, up to 44%, compared with primary hepatic tumors (0%).^32^ The situation was not the same for treatment of METS alone, because subgroup analyses included only 1 study following treatment of METS alone, with low statistical power.

Compared with RFA, cryoablation lacks an electrocautery needle tract, which represents the main possible risk for cryoablation-related bleeding. A previous study reported that bleeding was the major complication of cryoablation with average amount of blood loss of 700 ml.^36^ In our meta-analysis, the pooled rate of hemorrhage was 4.14% (18 of 435) in the cryoablation group and 1.16% (4 of 346) in the RFA group, showing a trend demonstrating the higher risk of bleeding regarding cryoablation compared with RFA despite a lack of statistical significance (OR = 1.92, 95% CI: 0.62–5.88). Regarding other complications data, our pooled analysis confirms significantly higher risks of thrombocytopenia and renal impairment in the cryoablation group than those in the RFA group. The complete coagulation of tumor and the surrounding hepatic microvasculature by RFA seems to prevent the rapid release of necrotic cellular products into the circulation and, thus, explains the lower risk of thrombocytopenia in the RFA patients, and renal dysfunction that has been reported after cryoablation.^37,38^

The strengths of the present meta-analysis are that we combine data from more studies than the previous one, including 1 multicenter RCT, thus giving greater statistical reliability, and no evidence of publication bias was found in all the analyses.

There are several limitations to this meta-analysis. Firstly, although we included more studies than the previous meta-analysis, the number of included studies is still limited, especially for the lack of RCTs. Secondly, we found statistical heterogeneity when we quantitatively pooled several outcomes. Although this was addressed by using random effects meta-analysis, subgroup analysis, and sensitivity analysis, these are unlikely to have fully accounted for heterogeneity. Thirdly, our inference is mostly based on observational studies, some included studies did not balance for other factors or only balance for a few important factors, thus, we cannot exclude the chance, residual or unmeasured confounding as alternative explanation of our findings. Fourthly, studies with newer devices for cryoablation, allowing an easy and safe percutaneous approach, are not available yet. Finally, the results of our meta-analysis were materially changed in some subgroup and sensitivity analyses, suggesting the results were not quite robust. In general, considering the limitations mentioned above, the physicians should interpret our results with adequate caution when they apply them in clinical practice.

## CONCLUSIONS

In conclusion, this meta-analysis suggests that the 2 methods had almost equal mortality of at least 6 months and did not show significant difference in local tumor progression according to both patients and tumors. However, compared with RFA, cryoablation showed significant higher risk of total complications, with increased risks of thrombocytopenia and renal impairment but not other complications. Given the relatively small studies and heterogeneity among studies, further large-scale, well-designed RCTs are urgently needed to identify the current findings and investigate the long-term effects of cryoablation compared with RFA for therapy of hepatic malignancies.

## References

[1] European Association for the Study of the Liver; European Organisation for Research and Treatment of Cancer. EASL-EORTC clinical practice guidelines: management of hepatocellular, carcinoma. *J Hepatol* 2012; 56:908–943. 22424438 10.1016/j.jhep.2011.12.001

[2] Curley SA Izzo F Delrio P Radiofrequency ablation of unresectable primary and metastatic hepatic malignancies: results in 123 patients. *Ann Surg* 1999; 230:1–8. 10400029 10.1097/00000658-199907000-00001 PMC1420837

[3] Gage AA Baust J Mechanisms of tissue injury in cryosurgery. *Cryobiology* 1998; 37:171–186. 9787063 10.1006/cryo.1998.2115

[4] Rubinsky B Lee CY Bastacky J The process of freezing and the mechanism of damage during hepatic cryosurgery. *Cryobiology* 1990; 27:85–97. 2311412 10.1016/0011-2240(90)90055-9

[5] McCarty TM Kuhn JA Cryotherapy for liver tumors. *Oncology (Williston Park)* 1998; 12:979–987.discussion 990, 993. 9684270

[6] Seifert JK Morris DL Prognostic factors after cryotherapy for hepatic metastases from colorectal cancer. *Ann Surg* 1998; 228:201–208. 9712565 10.1097/00000658-199808000-00009 PMC1191461

[7] Tandan VR Harmantas A Gallinger S Long-term survival after hepatic cryosurgery versus surgical resection for metastatic colorectal carcinoma: a critical review of the literature. *Can J Surg* 1997; 40:175–181. 9194777 PMC3952991

[8] Seifert JK Junginger T Morris DL A collective review of the world literature on hepatic cryotherapy. *J R Coll Surg Edinb* 1998; 43:141–154. 9654872

[9] Gervais DA Arellano RS Percutaneous tumor ablation for hepatocellular carcinoma. *Am J Roentgenol* 2011; 197:789–794. 21940565 10.2214/AJR.11.7656

[10] Rossi S Buscarini E Garbagnati F Percutaneous treatment of small hepatic tumors by an expandable RF needle electrode. *Am J Roentgenol* 1998; 170:1015–1022. 9530052 10.2214/ajr.170.4.9530052

[11] Rossi S Di Stasi M Buscarini E Percutaneous radiofrequency interstitial thermal ablation in the treatment of small hepatocellular carcinoma. *Cancer J Sci Am* 1995; 1:73–81. 9166457

[12] Siperstein AE Rogers SJ Hansen PD Laparoscopic thermal ablation of hepatic neuroendocrine tumor metastases. *Surgery* 1997; 122:1147–1154.discussion 1145–1154. 9426432 10.1016/s0039-6060(97)90221-x

[13] Rossi S Di Stasi M Buscarini E Percutaneous RF interstitial thermal ablation in the treatment of hepatic cancer. *Am J Roentgenol* 1996; 167:759–768. 8751696 10.2214/ajr.167.3.8751696

[14] Silverman SG Tuncali K Adams DF MR imaging-guided percutaneous cryotherapy of liver tumors: initial experience. *Radiology* 2000; 217:657–664. 11110925 10.1148/radiology.217.3.r00dc40657

[15] Orlacchio A Bazzocchi G Pastorelli D Percutaneous cryoablation of small hepatocellular carcinoma with US guidance and CT monitoring: initial experience. *Cardiovasc Intervent Radiol* 2008; 31:587–594. 18236104 10.1007/s00270-008-9293-9

[16] Tatli S Acar M Tuncali K Percutaneous cryoablation techniques and clinical applications. *Diagn Interv Radiol* 2010; 16:90–95. 19998248 10.4261/1305-3825.DIR.1922-08.0

[17] Stroup DF Berlin JA Morton SC Meta-analysis of observational studies in epidemiology: a proposal for reporting. Meta-analysis Of Observational Studies in Epidemiology (MOOSE) group. *JAMA* 2000; 283:2008–2012. 10789670 10.1001/jama.283.15.2008

[18] Higgins JP Thompson SG Deeks JJ Measuring inconsistency in meta-analyses. *BMJ* 2003; 327:557–560. 12958120 10.1136/bmj.327.7414.557 PMC192859

[19] Tobias A Assessing the influence of a single study in meta-analysis. *Stata Tech Bull* 1999; 47:15–17.

[20] Egger M Davey Smith G Schneider M Bias in meta-analysis detected by a simple, graphical test. *BMJ* 1997; 315:629–634. 9310563 10.1136/bmj.315.7109.629 PMC2127453

[21] Begg CB Mazumdar M Operating characteristics of a rank correlation test for publication bias. *Biometrics* 1994; 50:1088–1101. 7786990

[22] Bilchik AJ Wood TF Allegra D Cryosurgical ablation and radiofrequency ablation for unresectable hepatic malignant neoplasms: a proposed algorithm. *Arch Surg* 2000; 135:654–662.discussion 657–662. 10.1001/archsurg.135.6.657 10843361

[23] Joosten J Jager G Oyen W Cryosurgery and radiofrequency ablation for unresectable colorectal liver metastases. *Eur J Surg Oncol* 2005; 31:1152–1159. 16126363 10.1016/j.ejso.2005.07.010

[24] Pearson AS Izzo F Fleming RY Intraoperative radiofrequency ablation or cryoablation for hepatic malignancies. *Am J Surg* 1999; 178:592–599. 10670879 10.1016/s0002-9610(99)00234-2

[25] Tait IS Yong SM Cuschieri SA Laparoscopic in situ ablation of liver cancer with cryotherapy and radiofrequency ablation. *Br J Surg* 2002; 89:1613–1619. 12445075 10.1046/j.1365-2168.2002.02264.x

[26] Adam R Hagopian EJ Linhares M A comparison of percutaneous cryosurgery and percutaneous radiofrequency for unresectable hepatic malignancies. *Arch Surg* 2002; 137:1332–1339.discussion 1340. 12470093 10.1001/archsurg.137.12.1332

[27] Dunne RM Shyn PB Sung JC Percutaneous treatment of hepatocellular carcinoma in patients with cirrhosis: a comparison of the safety of cryoablation and radiofrequency ablation. *Eur J Radiol* 2014; 83:632–638. 24529593 10.1016/j.ejrad.2014.01.007

[28] Wang C Wang H Yang W Multicenter randomized controlled trial of percutaneous cryoablation versus radiofrequency ablation in hepatocellular carcinoma. *Hepatology* 2015; 61:1579–1590. 25284802 10.1002/hep.27548

[29] Ravikumar TS Kane R Cady B A 5-year study of cryosurgery in the treatment of liver tumors. *Arch Surg* 1991; 126:1520–1523.discussion 1523–1524. 1842183 10.1001/archsurg.1991.01410360094015

[30] Seifert JK Achenbach T Heintz A Cryotherapy for liver metastases. *Int J Colorectal Dis* 2000; 15:161–166. 10954187 10.1007/s003840000221

[31] Crews KA Kuhn JA McCarty TM Cryosurgical ablation of hepatic tumors. *Am J Surg* 1997; 174:614–617.discussion 617–618. 9409584 10.1016/s0002-9610(97)00179-7

[32] Adam R Akpinar E Johann M Place of cryosurgery in the treatment of malignant liver tumors. *Ann Surg* 1997; 225:39–50. 8998119 10.1097/00000658-199701000-00005 PMC1190603

[33] Wood TF Rose DM Chung M Radiofrequency ablation of 231 unresectable hepatic tumors: indications, limitations, and complications. *Ann Surg Oncol* 2000; 7:593–600. 11005558 10.1007/BF02725339

[34] Curley SA Izzo F Ellis LM Radiofrequency ablation of hepatocellular cancer in 110 patients with cirrhosis. *Ann Surg* 2000; 232:381–391. 10973388 10.1097/00000658-200009000-00010 PMC1421151

[35] Elias D Goharin A El OA Usefulness of intraoperative radiofrequency thermoablation of liver tumours associated or not with hepatectomy. *Eur J Surg Oncol* 2000; 26:763–769. 11087642 10.1053/ejso.2000.1000

[36] Wong WS Patel SC Cruz FS Cryosurgery as a treatment for advanced stage hepatocellular carcinoma: results, complications, and alcohol ablation. *Cancer* 1998; 82:1268–1278. 9529018 10.1002/(sici)1097-0142(19980401)82:7<1268::aid-cncr9>3.0.co;2-b

[37] Ross WB Horton M Bertolino P Cryotherapy of liver tumours—a practical guide. *HPB Surg* 1995; 8:167–173. 7547619 10.1155/1995/93283 PMC2423787

[38] Cuschieri A Crosthwaite G Shimi S Hepatic cryotherapy for liver tumors. Development and clinical evaluation of a high-efficiency insulated multineedle probe system for open and laparoscopic use. *Surg Endosc* 1995; 9:483–489. 7676367 10.1007/BF00206832

